# Proliferative Glomerulonephritis: Risk Factor for Hypertension in Lupus

**DOI:** 10.1155/2021/6691821

**Published:** 2021-04-15

**Authors:** Mansour Mbengue, Motula Latou Lot, Seynabou Diagne, Abdou Niang

**Affiliations:** ^1^Department of Nephrology, Dalal Jamm University Hospital, Dakar, Senegal; ^2^Department of Nephrology, Aristide Le Dantec University Hospital, Dakar, Senegal; ^3^Department of Nephrology, Pikine Hospital, Dakar, Senegal

## Abstract

Studies report a high prevalence of hypertension in lupus, reaching up to 74%. The incidence of hypertension in SLE patients is increased with the severity of the kidney damage. This work was carried out with the objective of determining the prevalence of hypertension in lupus nephritis and to seek the existence of an association between the presence of a proliferative glomerulonephritis and hypertension. *Patients and Methods.* This was a case-control study, carried out in the nephrology department of the Aristide Le Dantec University Hospital in Dakar. All records of patients with lupus nephritis over a 10-year period, from January 01, 2007, to December 31, 2016, were included. *Results*. During the study period, out of 64 lupus nephritis records collected, 28 patients had hypertension, for a hospital prevalence of 43.75%. The mean age of the patients was 30.64 years ± 10.44. There were 24 women and 4 men. The mean systolic blood pressure was 156 mmHg (110–220) and the mean diastolic blood pressure was 100 mmHg (80–130). The mean serum creatinine was 29.48 mg/l ± 24.99. The mean proteinuria was 4.50 g/24 h ± 2.87. Hypertriglyceridemia was observed in one patient. Hypercholesterolemia was present in 3 patients. HDL levels were normal in all patients and elevated LDL levels were noted in all 4 patients. None of our patients had diabetes. Class III was found in 11 cases, class IV in 14 cases, pure class V in 2 cases, and class II in 1 case. Hypertension was associated with the presence of proliferative glomerulonephritis (odds ratio, 7.45; 95% CI, 1.9 to 29.1; *p*=0.002). *Conclusion*. Hypertension is common in lupus nephritis. The presence of a proliferative glomerulonephritis is a risk factor for the development of arterial hypertension. Screening and adequate management of hypertension are essential for the prevention of the progression of chronic kidney disease in lupus.

## 1. Introduction

Several studies report a high frequency of hypertension in lupus, reaching up to 74% in some cohorts [1–4]. The incidence of hypertension in SLE patients is also increased with the severity of the kidney damage. Besides nephropathy, other factors are involved in the development of hypertension in lupus. These include activation of the renin angiotensin aldosterone system (RAAS) and immune dysregulation seen in patients with SLE. The presence of hypertension has several implications for the prognosis of lupus nephritis. Studies in the recent years have shown that the primary cause of death for SLE patients is no longer the autoimmune pathology itself, neither the infectious consequences of immunosuppressive treatments, but primarily cardiovascular pathologies [5].

Lupus is the cause of major and accelerated atherosclerosis, possibly through nephropathy but also through chronic inflammation and metabolic complications, immunosuppressive treatments, and corticosteroids. A recent meta-analysis clearly confirms that SLE patients have a cardiovascular risk almost twice that of the general population, this being much more marked for the youngest patients [6].

Hypertension is one of the important determinants of the progression of atherosclerosis in lupus. In addition, hypertension is recognized as one of the factors contributing to the progression of chronic kidney disease (CKD). Hypertension in lupus nephritis is not well described in sub-Saharan Africa. This work is carried out with the objective of determining the prevalence of hypertension in lupus nephritis and to seek the existence of an association between the presence of a proliferative glomerulonephritis and hypertension.

## 2. Patients and Methods

This was a case-control study carried out in the nephrology department of the Aristide Le Dantec University Hospital in Dakar. All records of patients with lupus nephritis over a 10-year period, from January 01, 2007, to December 31, 2016, were included. The cases were chosen by taking all the files of patients who presented hypertension. The patients who did not have hypertension were the controls. Patients who had a proliferative glomerulonephritis were the exposed. The patients who did not present a proliferative glomerulonephritis were the unexposed. The diagnosis of lupus nephritis was made after a concordant renal puncture biopsy associated with proteinuria greater than 0.5 g/24 h or with active urine sediment [7]. Proliferative glomerulonephritis was defined by classes III or IV associated or not with a class V according to the classification of the ISN/RPS of 2003 [7]. Hypertension was defined according to the definition of the World Health Organization [8]. For each patient retained, epidemiological, clinical, biological, histological, therapeutic, and evolutionary data were studied. Glomerular filtration rate (GFR) was estimated according to the MDRD formula (The Modification of Diet in Renal Disease). The evolving modalities were recorded after a 6-month follow-up. Complete or partial remission, relapse, and resistance were defined according to the 2012 European League against Rheumatism and European Renal Association, European Dialysis and Transplant Association (EULAR/ERA/EDTA) criteria [9]. The data were collected on a preestablished form. They were entered with Sphinx software version 5.1.0.2. The data analysis was carried out with the SPSS software (Statistical package for Social Sciences) version 18. The descriptive study was carried out with the calculation of the frequencies and proportions for the qualitative variables and the calculation of the means, standard deviations, for the quantitative variables. The analytical study was done with cross tables. To compare the frequencies, we used Pearson's chi-square test or Fisher's two-tailed exact test; according to their conditions of applicability, the comparison of means was made with the analysis of variance test with a threshold of significance *p* < 0.05. The 95% confidence interval of the odds ratio was calculated using the semi-exact method.

## 3. Results

During the study period, out of 64 lupus nephritis records collected, 28 patients were included, for a hospital prevalence of 43.75%. The mean age of the patients was 30.64 years ±10.44. There were 24 women and 4 men at a sex ratio of 0.16. The mean systolic blood pressure was 156 mmHg (110–220) and the mean diastolic blood pressure was 100 mmHg (80–130). The mean serum creatinine was 29.48 mg/*l* ± 24.99. The mean proteinuria was 4.50 g/24h ± 2.87. Hypertriglyceridemia was observed in one patient. Total hypercholesterolemia was present in 3 patients. HDL levels were normal in all patients and elevated LDL levels were noted in all 4 patients (Table 1). None of our patients had diabetes. Class III was found in 11 cases, class IV in 14 cases, pure class V in 2 cases, and class II in 1 case. In the induction treatment of patients with proliferative glomerulonephritis, cyclophosphamide was used in 12 cases, mycophenolate mofetil in 4 cases, and azathioprine in 1 case. This immunosuppressive treatment was associated with corticosteroid therapy in all cases. In the patient with class V + II, cyclophosphamide, combined with corticosteroid therapy, was used. After 6-month follow-up, there were 5 lost to follow-up. Of the remaining 23 patients, 6 were in complete remission, 6 were in partial remission, and 11 were resistant. There are 5 patients who had developed end-stage renal disease (ESRD). Death was observed in 4 patients and the causes were pulmonary embolism in one patient, bacterial meningitis in one patient, and pulmonary *tuberculosis* in one patient. Hypertension was associated with the presence of proliferative glomerulonephritis (odds ratio, 7.45; 95% CI, 1.9 to 29.1; *p*=0.002) (Table 2).

## 4. Discussion

The association of lupus nephritis and hypertension is well reported [10, 11]. Several studies have reported the high incidence of hypertension in lupus as high as 74% [1–4]. In our series, 43.75% of patients had hypertension. In sub-Saharan Africa, studies found a prevalence ranging from 30% to 29.3% [12, 13]. On the other hand, the prevalence of hypertension was higher in the series of Contreras et al. [14] with 70% of cases, of Aoudia et al. [8] with 48.85% of cases, and of Martin-Gomez et al. [15] with 54.1% of cases. Based on these results, it was found that the prevalence of hypertension in our study was lower than in studies done in white patients. This fact deserves a questioning because hypertension in general and particularly in patients with glomerulopathy is more frequent in black people because of the genetic predisposition linked to the presence of a polymorphism of the Apol 1 gene which codes for the apolipoprotein 1. More studies are needed to answer this question. This hypertension is multifactorial; glomerular involvement of lupus is the basis; it is worsened in the event of associated renal failure, especially since the patient is on corticosteroid therapy [15].

The incidence of hypertension in patients with lupus nephritis was also increased with both the severity of the kidney damage and the loss of kidney function. In our study, there is an association between hypertension and the presence of a proliferative glomerulonephritis. Okpechi et al. had also found this association between hypertension and the existence of a proliferative glomerulonephritis [13]. These results could be explained by the fact that cell proliferation leads to a decrease in GFR which will lead to renal failure and a decrease in urine output. The hypertension would be volo-dependent in relation to the hydrosodium retention linked to the decrease in urine output and to the reduction in oncotic pressure linked to hypoalbuminemia. Because of the kidney's importance in the long-term control of blood pressure, kidney damage may contribute to the development of hypertension in patients with SLE. Impaired renal hemodynamics and tubular function are the contributing factors since the kidney's ability to excrete sodium and water in response to changes in blood pressure is essential for effective long-term blood pressure control. Renal vascular dysfunction is a factor that may promote impaired renal hemodynamics. In addition, several studies have shown endothelial vascular dysfunction in lupus and impaired endothelial-dependent vasodilation and endothelial repair mechanisms in lupus patients [16].

However, hypertension associated with lupus can occur independently of the nephropathy [17]. For example, a recent study by Shaharir et al. found that 53% of patients with lupus in one cohort had hypertension despite the absence of renal impairment [18]. Other factors are involved in the onset of hypertension in lupus, including RAAS and immune dysregulation.

RAAS is also implicated because of its role in blood pressure balance and homeostasis. Angiotensin II can stimulate the production of endothelin-1 (ET-1), a peptide whose role in blood pressure control results from a balance between its vasoconstrictor and natriuretic actions in the renal medulla. Plasma ET-1 levels are increased in patients with lupus [19], and serum from lupus patients can induce ET-1 production by endothelial cells in vitro [20].

It is widely recognized that immune dysregulation and chronic inflammation have a role in the pathophysiology of hypertension in lupus. [21]. This is supported by the fact that treatment with the immunosuppressant mycophenolate mofetil in both animal models and in patients with essential hypertension reduced blood pressure [22]. Various inflammatory cytokines, including interleukins (IL-6, IL-17, and IL-18), type I interferons, and tumor necrosis factor-*α* (TNF*α*) have been implicated in the pathogenesis of lupus. Likewise, studies report a correlation between cytokines such as IL-6, TNF*α*, and C-reactive protein and blood pressure in patients with essential hypertension [23]. Inflammatory cytokines interact with important blood pressure regulators such as RAAS [24] and the sympathetic nervous system [25].

## 5. Conclusion

Hypertension is common in lupus nephritis. The presence of a proliferative glomerulonephritis is a risk factor for the onset of hypertension. Screening and adequate management of hypertension are essential for preventing the progression of chronic kidney disease in Lupus.

### 5.1. Limits

The limitation of our study was the lack of match on age and sex ratio between cases and controls. But we can see in our study that the cases and controls were comparable on age. Corticosteroid therapy could not be a confounding factor as hypertension was diagnosed before the patients were started on corticosteroids.

## Figures and Tables

**Table 1:** Comparison of socio-demographic, clinical characteristics of cases and controls.

Variables	Hypertension (*n* = 28)	Without hypertension (*n* = 36)	*p* value
Mean age (years)	30.64 ± 10.45	32.51 ± 11.65	0.51
Sex			0.42
Women	24 (85.70%)	28 (77.70%)	
Men	4 (14.30%)	8 (22.30%)	
Serum creatinine (mg/L)	29.48 ± 24.99	11.61 ± 12.47	0.01
Hemoglobin level (g/dL)	8.80 ± 2.48	12.55 ± 17.43	0.27
Calcemia (mg/L)	87.73 ± 8.70	91.35 ± 7.80	0.20
Phosphoremia (mg/L)	56.50 ± 38.78	56.50 ± 38.78	0.32
Albuminemia (g/L)	27.20 ± 30.76	22.21 ± 7.74	0.46
Protidemia (g/L)	57.23 ± 9.47	54.21 ± 13.65	0.42
Total cholesterol (g/L)	2.95 ± 0.61	2.30 ± 0.53	0.13
HDL cholesterol (g/L)	0.56 ± 0.10	0.52 ± 0.20	0.75
LDL cholesterol (g/L)	2.04 ± 0.48	1.19 ± 0.72	0.09
Triglycerides (g/L)	1.42 ± 0.51	1.35 ± 0.84	0.89
Proteinuria (g/24 h)	4.50 ± 2.87	3.61 ± 3.24	0.26
Anti-DNA antibodies (%)	4 (36.40)	6 (50.0)	0.40
GFR (ml/min/1.73 m^2^)	52.42 ± 40.68	121.48 ± 59.60	

eGFR = estimated glomerular filtration rate according to 4-variable MDRD equation; HDL = high density lipoproteins; LDL = low density lipoproteins.

**Table 2:** Cross table of cases and controls.

	Hypertension	Without hypertension	Total
Proliferative glomerulonephritis	25	19	44
Without proliferative glomerulonephritis	3	17	20
Total	28	36	64

Odds ratio, 7.45; 95% CI, 1.9 to 29.1; *p*=0.002.

## Data Availability

The data used to support the findings of this study are included within the article.
